# Avoidance-related behavioral and blood-based physiological responses of Nguni and Boran cattle subjected to routine handling activities post relocation

**DOI:** 10.3389/fvets.2023.1188505

**Published:** 2023-07-06

**Authors:** Mhlangabezi Slayi

**Affiliations:** Risk and Vulnerability Science Centre, University of Fort Hare, Alice, South Africa

**Keywords:** bovine serum, cattle welfare, chute score, farm workers, fear response, local breeds

## Abstract

**Introduction:**

This study aimed to investigate the avoidance-related behavioral and blood-based physiological responses of Nguni and Boran cattle during routine handling activities post-relocation, with a particular focus on the effect of breed, week, and waiting time.

**Methods:**

A total of 20 animals, 10 from each breed, were subjected to handling activities at fortnight intervals post-relocation. The animals were observed for entry time (ES), chute score (CS), kicking score (KS), blood sampling time, cortisol, and glucose concentrations. The data were analyzed using ANOVA and regression analysis.

**Results and Discussion:**

Results showed that breed had a significant effect on avoidance-related behavioral responses (ES: *p* = 0.0032; CS: *p* = 0.0071; and EX: *p* = 0.0320), with Nguni cattle displaying more active avoidance behaviors compared to Boran cattle. Additionally, breed differences were observed in physiological responses, with Nguni cattle exhibiting higher cortisol and glucose levels compared to Boran cattle. Waiting time in the race had a greater impact on chute score (CS: *p* = 0.0037) and cortisol release (*p* = 0.0375) in the two breeds. Regression analysis revealed that the amount of time spent in the handling facility prior to sampling and the duration of blood collection significantly increased from week 3 to 15. Steers that waited in the race for more than 10 min had higher cortisol levels (*p* = 0.0031). These findings suggest that breed-specific management practices may be necessary to reduce stress-related responses and improve animal welfare during routine handling activities post-relocation. Overall, this study highlights the importance of considering the effects of breed, week, and waiting time when evaluating the avoidance-related behavioral and blood-based physiological responses of cattle during routine handling activities. These factors play a significant role in understanding and addressing the stress and welfare concerns associated with handling procedures, particularly after relocation.

## 1. Introduction

Performing routine husbandry practices such as vaccination, weighing, dipping and dosing require handling of livestock, which is a common practice in livestock production (1). This process usually involves moving cattle through various handling facilities with the help of farm workers (2). This interaction between humans and animals can result in the isolation of animals from the herd, and the workers involved in handling the cattle often have limited training (3). Despite the limited training of beef cattle workers, research indicates that animals respond to human presence with either a flighty or aggressive behavior (4). This change in behavior is influenced by the animal’s previous experience with handling procedures, which can trigger the secretion of hormones and neurons, leading to stress in the animal (5, 6). In such situations, the animal may react inappropriately, causing harm or injury to itself, its handler, and the handling facilities on the farm (7).

Animal handling difficulties can result in negative human behaviors such as frustration and abuse, as reported by several researchers (8). These behaviors can lead to injuries, damaged facilities, reduced productivity, and economic losses, particularly in small-holder farming systems (9, 10). Despite the significant impact on profitability, this problem still persists. To improve human-animal interactions, positive handling techniques such as frequent contact between stockman and animals have been suggested (11). Improving corral handling facilities through the adoption of advanced technologies has also been proposed (12). However, the effectiveness of these methods in grass-fed or pasture-based beef cattle systems has not been thoroughly explored. Extensively raised cattle tend to react more negatively to handling procedures than other livestock species, such as dairy cattle, which are frequently handled (13). Therefore, it is widely accepted that handling extensively raised cattle can be more challenging and dangerous, particularly those with more excitable temperaments (14).

Numerous studies have examined animal responses to human interactions in different scenarios, with the aim of addressing handling issues and determining which animals should not be selected for breeding programs (4, 12). Understanding cattle temperament has practical and economic implications, as different breeds react differently to human interactions during farming operations (2). However, much of the research in this area has focused on exotic cattle breeds, such as Brahman and Simmental, which are often handled regularly (15, 16). This is problematic because a significant proportion of beef cattle breeds in South Africa and other developing countries are indigenous and extensively managed with limited human contact. Nguni and Boran cattle are two important breeds that are widely used in sub-Saharan Africa for meat and dairy production (17, 18). However, little is known about the avoidance behavior and blood-based responses of these cattle to handling activities post-relocation. Relocation of cattle can be a significant source of stress due to changes in the social and physical environment (17), making it a crucial factor to consider when assessing the stress response of cattle during handling activities. Investigating the avoidance behavior and blood-based responses of Nguni and Boran cattle to handling activities post-relocation can provide valuable insights into the factors that contribute to stress and help identify effective handling strategies to improve animal welfare.

The hypothesis for this study is that relocation and breed will have a significant effect on avoidance behavior and blood-based physiological responses of Nguni and Boran cattle during handling activities. We expect that the relocation of cattle to a new environment will result in increased avoidance behavior and elevated cortisol and glucose levels in their blood. We also anticipate that Nguni and Boran cattle may have different responses to handling activities due to their genetic differences, resulting in breed-specific avoidance behavior and blood-based physiological responses. By investigating these hypotheses, we aim to provide valuable insights into the stress responses of Nguni and Boran cattle during handling activities, highlighting the importance of minimizing stress to improve animal welfare and enhance the efficiency of handling procedures.

## 2. Materials and methods

### 2.1. Ethical clearance

The accommodation and care of animals in this study adhered to ethical guidelines and regulations. Prior to conducting the study, permission was obtained from the University of Fort Hare Animal Research Ethics Committee (MUC551SSLA01). This committee ensures that all animal-related research is conducted in a humane and responsible manner, taking into consideration the well-being and welfare of the animals involved. The committee reviews and approves research protocols, ensuring compliance with ethical standards and guidelines for the proper accommodation, handling, and care of animals. By obtaining the necessary ethical clearance, this study demonstrates its commitment to upholding ethical principles and ensuring the welfare of the animals throughout the research process.

### 2.2. Animal origin and experimental site description

**Figure 1:**
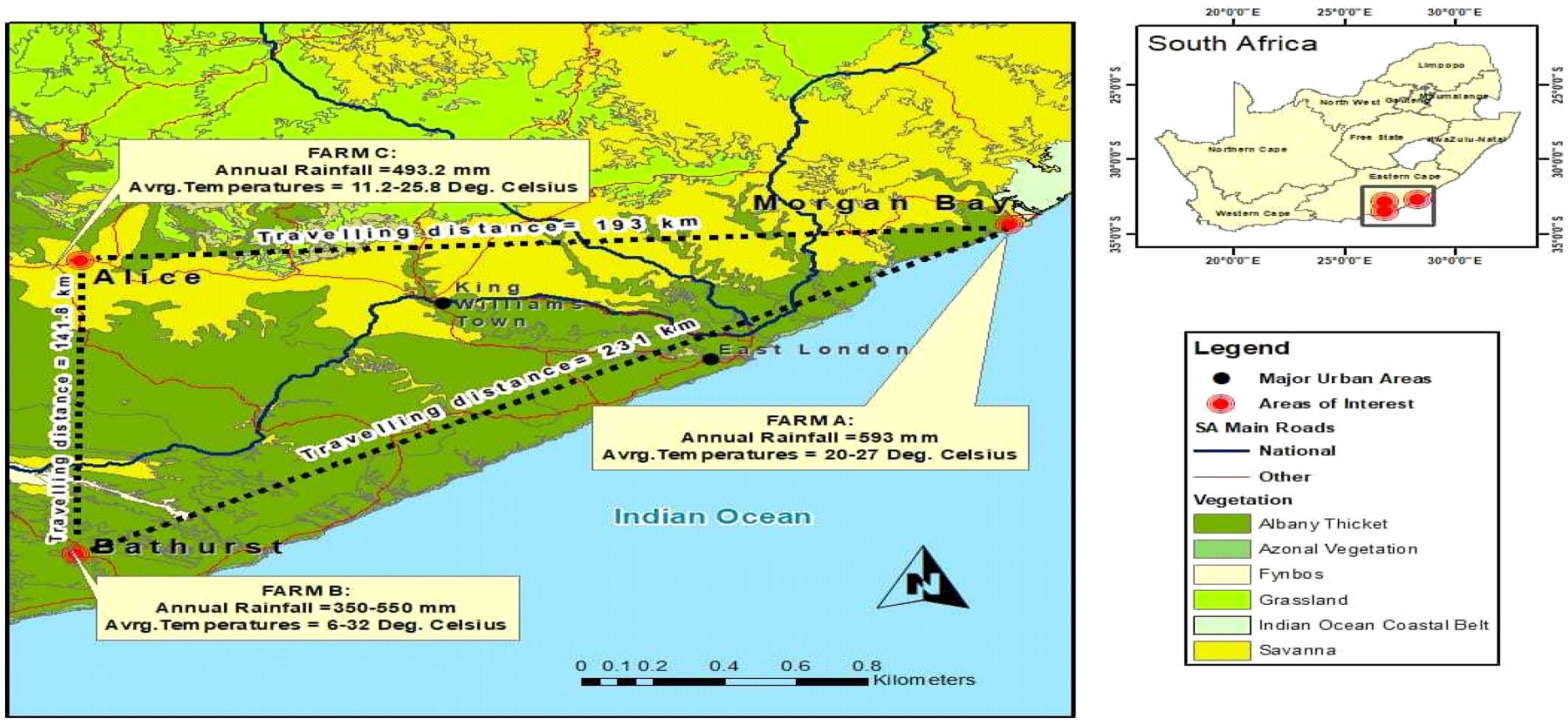
Map showing the geographic location of the study site.

The animals used in the study were obtained from two stud breeders located in Bathurst and Morgan Bay, and then transported to the Honeydale Research farm, which belongs to the University of Fort Hare in Alice, South Africa (as shown in Figure 1). These farms are renowned for breeding and producing high-quality Nguni and Boran cattle. The Nguni cattle, in particular, are known for their ability to adapt to diverse and challenging environmental conditions due to their smaller body frame, which makes them more resilient to extreme temperatures (19). Additionally, they possess a strong immunity to tick-borne illnesses and helminthosis (20). Mature Nguni bulls usually weigh between 500 and 600 kg and exhibit well-developed, muscular cervicothoracic humps in front of the foreleg, while Nguni cows typically weigh between 300 and 400 kg and have a distinctive sloping rump, which aids in calving (21). Boran cattle, on the other hand, are a medium-sized beef breed that can be gray, fawn, or red in color (3). They are well-known for their high fertility, good mothering ability, excellent temperament, and great survivability in harsh conditions (22). They also possess good meat quality and early maturity, making them a valuable resource in crossbreeding programs aimed at enhancing the productivity of beef herds (23). As mentioned earlier, the animals used in the study were obtained from two farms located in Morgan Bay and Bathurst. Subsequently, they were transported to Honeydale Farm in Alice, which served as the designated study site. The experimental area is situated at 32.8° latitude and 26.9° longitude, approximately 520 m above sea level. It has a semi-arid climate, with a mean annual temperature of 28.7°C and a mean annual rainfall of 453 mm. The experimental site spans 210 ha and is divided into 36 paddocks, each measuring 5.84 ha. The grazing patterns of the cattle in these paddocks are highly heterogeneous, with some areas displaying signs of degradation and encroachment. The soils in the area are mostly shallow, with a depth of less than 450 mm, and are comprised of deep alluvial-derived types in arable lands (24). The region is characterized by steep, isolated mountains and hills, with several dams and water streams. The vegetation consists of numerous tree, shrub, and grass species, with Vachelia karroo, *Themeda triandra*, *Digitaria eriantha*, Eragrostis spp., and *Pennisetum clandestinum* being the dominant plant species (25). The ecological area and veld type predominantly belong to the Bhisho Thornveld, as classified by Acocks (26).

### 2.3. Animal management

An experienced veterinarian with more than 30 years professional experience clinically examined and selected a total of 20 steers, comprising 10 Nguni and 10 Boran, aged 9 months, to ensure their health fitness prior to their use in the study. Upon their arrival at the Honeydale Research Farm, the steers were supplemented with Vitamin B complex and inoculated with Blanthrax^®^ to protect against Anthrax and other related diseases that are known to be prevalent in the area. Irrespective of their source of origin, the steers were allocated to a single paddock (500 × 500 m per paddock, length × width) to allow acclimatization and to establish social relationships with their new herd mates. A 6-month resting period was applied to each paddock to ensure that there was sufficient forage before introducing the animals. A 24-h accessible watering system was centrally located within each paddock. To enable identification, different numbered and colored ear tags were fitted on each animal, with green for Boran and white for Nguni. An individual code was marked on the back of each steer using washable dye to differentiate between animals with the same body color during behavioral observations. The steers were dipped with DRASTIC DEADLINE^®^ fortnightly to control tick infestation in the area. Throughout the trial period, the steers had free access to water and were allowed to free-range together. Feeding the animals was done through rotational grazing, with the steers moved to a new paddock after every 14 days. The study utilized a total of six paddocks to house the animals over 15 weeks trial period.

### 2.4. Data collection

A plan was organized with the farm manager and stock personnel to gather the animals as a single group from the grazing pastures to the holding facility every fortnight for a period of 15 weeks, at 7:00 in the morning. Upon arrival at the handling facilities, the animals were guided into an 8 × 6 m collecting pen, which had a 3 m wide entrance for easy access. From there, the animals were directed through a funnel-shaped forcing pen to the race. The angle of the funnel was set at 30° to ensure that the cattle did not block or turn at the mouth of the race, or become jammed at the race entrance. The race was 9 m in length and could accommodate 10 animals at a time before being taken to the chute. The internal width of the race was 650 mm, and its height was 1.5 m above floor level, with a non-slip concrete surface. Blood sampling and chute scoring were conducted on each animal as they entered the holding crush, which was located at the end of the race. Behavioral scoring was carried out when each animal was separated from the rest of the group and allowed to move voluntarily through the race, single-file chute, and holding crush. The steers had direct visual contact with the preceding animal that was restrained in the chute. The time spent waiting in the race for each animal to be blood sampled was also recorded, with waiting durations categorized into three periods based on the amounts of time spent (less than 5, 6–10, and more than 10 min, respectively). Humane interventions were applied when animals displayed agitation or avoidance behavior to enter or exit the chute, using low-stress handling techniques such as guiding the animals with flags or paddles instead of shouting or hitting them. In some instances, the observer moved slowly and calmly to avoid startling the animals. After behavioral scoring and blood sampling were completed, the animals were returned together to the pasture.

#### 2.4.1. Behavioral scoring

A single trained observer was responsible for recording the behavior of each individual animal in the holding crush throughout the study. The observer recorded the entry time, chute score, blood sampling time, kicking score, and exit time for each animal. The chute score and kicking score for each animal are presented in Table 1. The same observer who evaluated the chute score collected all blood samples. The elapsed time required for each animal to enter, be blood sampled, and exit the chute was measured in minutes using a stopwatch. The scoring system used to evaluate behavior is similar to methods described in previous studies (12, 27, 28). Table 1 provides details of the chute scores and kicking behavior scores observed during handling. Higher scores indicate better behavior, with the chute score being based on a 1–5 point scale. The behavior of the animals as they pass through the chute is observed to determine the chute score, which is assigned based on the criteria listed below. Higher scores indicate greater kicking or struggling behavior. The kicking score is also based on a 1–5 point scale, and once the animals are restrained, their behavior is evaluated, and a score is assigned based on the recommendations provided below. The scores for kicking and chute behavior can also be used to assess the effectiveness of various handling techniques.

**Table 1:** Description of chute and kicking behavior scores.

Chute	Kicking behavior
1: Cattle refuse to enter the chute and become highly agitated.	1: Cattle kick or struggle constantly and require extreme force to restrain them.
2: Cattle enter the chute but require excessive prodding or pushing.	2: Cattle kick or struggle frequently and require strong physical restraint.
3: Cattle enter the chute without much resistance but require some prodding or nudging.	3: Cattle kick or struggle occasionally and require moderate physical restraint.
4: Cattle enter the chute willingly but require a small amount of prodding or guidance.	4: Cattle rarely kick or struggle and require minimal physical restraint.
5: Cattle enter the chute calmly and without hesitation.	5: Cattle do not kick or struggle at all during handling.

#### 2.4.2. Blood sampling and analysis

To obtain blood samples from the animals, a chute and halter were used for individual restraint and this enabled easier handling. A skilled veterinarian collected the samples using a 20-gauge vacutainer needle per animal. The blood samples were obtained from the jugular vein and collected in heparinized tubes. After sampling, the tubes were immediately placed on ice and transported to the laboratory for processing. The samples were then centrifuged at 3,000 rpm for 10 min to separate the plasma from the blood cells. The resulting plasma was transferred to sterile 5-ml polypropylene tubes and frozen at −20°C until they were assayed to determine the concentration of cortisol using a radioimmunoassay method described by Curley et al. (5). Each sample was determined in duplicate, and the results were expressed in ng/ml along with corresponding controls. The variation coefficients of the analysis, inter-assay and intra-assay were 7 and 8%, respectively. The serum samples were also analyzed to determine the concentration of glucose (mmol/L) using a Multichannel Technicon Analyzer (RA500) and reagents from Bayer diagnostics (SA).

### 2.5. Statistical analysis

Descriptive statistics, including means and standard errors, were calculated for avoidance behavioral and blood based physiological variables. Statistical analysis (29) was conducted using ANOVA and multiple regression to determine significant differences between the breeds, week and waiting time. A *p*-value of less than 0.05 was considered statistically significant. The statistical model used for the analysis is as follows:

Y = α + β1Breed + β2Week + β3Waiting Time + β4Interference + β5Breed × Week + β6Breed × Waiting Time + β7Breed × Interference + β8Week × Waiting Time + β9Week × Interference + β10Waiting Time × Interference + u + ε.

In this model, Y represents the response variable (entry time, chute score, sampling time, glucose levels, or cortisol levels), α represents the intercept, and β1-β10 represent the fixed effects coefficients for Breed, Week, Waiting Time, Interference, and their interactions, respectively. In this equation, “u” represents the random effects associated with the animal factor. The random effects account for the variability among different animals that cannot be explained by the fixed effects variables. The error term ε still represents the residual variability in the model. The mixed-effects model allows for capturing both the fixed effects (β coefficients) and the random effects (u) associated with the animal factor, providing a more comprehensive analysis of the data. The variable ε represents the residual error. The inclusion of interaction terms in the model allowed us to examine how the effect of one variable changes with respect to the other variables in the model. For example, the interaction between Breed and Week allowed us to determine whether the effect of Week on the response variable is different for Nguni and Boran cattle. The multiple regression analysis was used to estimate the coefficients and test the statistical significance of each variable and interaction term. This analysis provided insights into the relationships between the predictor variables (Breed, Week, Waiting Time, Interference) and the response variables (entry time, chute score, kicking score, sampling time, exit time, glucose levels, and cortisol levels) and helps determine the significance of these effects in the study.

## 3. Results

### 3.1. Behavioral responses and handling pressure required to restrain Nguni and Boran cattle

Table 2 shows the mean values for different parameters measured in Boran and Nguni cattle over a period of 15 weeks, along with their standard errors and value of ps. There is a significant main effect of breed (B) on entry time (*p* = 0.0032), indicating that the breed of the animal influences the time it takes for entry. However, there is no significant interaction effect between breed and period (*p* = 0.8142). At the same time, there is a significant main effect of period (*p* = 0.0071) on chute score, suggesting that the period of weeks affects the score. Additionally, there is a significant interaction effect between breed and period (*p* = 0.0376), indicating that the relationship between breed and period is not consistent across all time points. There is a significant main effect of period (*p* < 0.0001) on sampling time, meaning that the time taken for sampling is influenced by the period of weeks. Moreover, there is a significant interaction effect between breed and period (B × *p* < 0.0001), indicating that the effect of breed on sampling time varies across different time points. There are no significant main effects of breed or period on kicking score (*p* > 0.05). Additionally, there is no significant interaction effect between breed and period (*p* = 0.5215), suggesting that the relationship between breed and period does not significantly affect kicking score. There is a significant main effect of period (*p* = 0.0320) on exit time, indicating that the period of weeks influences the time it takes for animals to exit. However, there is no significant main effect of breed or interaction effect between breed and period on exit time (*p* > 0.05), suggesting that breed does not significantly affect exit time.

**Table 2:** LSMeans ± SE for the behavioral scores of Nguni and Boran cattle during blood collection according to sampling weeks.

	Breed	Period (weeks)								±SEM	*p*		
		Week 1	Week 3	Week 5	Week 7	Week 9	Week 11	Week 13	Week 15				
											B	P	B × P
Entrytime (minutes)	Boran	1.97^c,y^	2.11^c,y^	3.50^b,y^	4.05^b,y^	4.51^a,y^	4.85^a,x^	5.03^a,x^	4.52^a,x^	0.30	0.0032	<0.0001	0.8142
	Nguni	2.61^b,x^	2.99^b,x^	4.33^a,x^	4.57^a,x^	5.11^a,x^	5.02^a,x^	4.95^a,x^	4.92^a,x^				
Chute score	Boran	3.24^b,x^	3.53^b,x^	4.22^a,x^	4.10^a,x^	3.63^b,x^	3.82^b,x^	3.61^b,x^	3.80^b,x^	0.29	0.0071	0.7318	0.0376
	Nguni	3.71^a,x^	3.94^a,x^	3.35^a,y^	2.81^b,y^	3.33^a,y^	2.91^b,y^	3.32^a,y^	3.22^a,y^				
Sampling time (s)	Boran	1.08^b,x^	1.20^b,x^	1.36^b,y^	1.94^a,x^	1.77^a,x^	1.58^a,x^	2.33^a,x^	1.77^a,x^	0.20	0.5286	<0.0001	<0.0001
	Nguni	1.18^b,x^	1.18^b,x^	1.64^a,x^	1.85^a,x^	1.68^a,x^	1.70^a,x^	2.25^a,x^	1.89^a,x^				
Kicking score	Boran	1.30^b,y^	1.50^b,y^	2.00^a,x^	1.70^b,x^	1.70^b,x^	2.20^a,x^	2.10^a,x^	2.20^a,x^	0.23	0.3174	0.0581	0.5215
	Nguni	1.90^a,x^	2.10^a,x^	2.10^a,x^	1.90^a,x^	2.20^a,x^	2.10^a,x^	2.30^a,x^	2.30^a,x^				
Exit time (minutes)	Boran	0.49^a,x^	0.43^a,x^	0.34^b,x^	0.31^b,x^	0.33^b,x^	0.30^b,x^	0.28^b,x^	0.23^b.x^	0.04	0.0320	0.0001	0.2301
	Nguni	0.35^a,y^	0.29^a,y^	0.24^b,x^	0.33^a,x^	0.24^b,x^	0.30^a,x^	0.21^b,x^	0.24^b,x^				

^a–c^Different letters in the same row are significantly different (*P* ≤ 0.05). ^x,y^Column means with different letters within each variable are significantly different (*p* < 0.05). B, Breed; P, Period; B × P, Breed × Period interaction; SEM, Standard Error of the Mean.

### 3.2. Type of human assistance required to pursue the Nguni and Boran cattle to enter and exit the handling facility

Table 3 shows the frequency and percentage of the type of human assistance required to pursue Nguni and Boran cattle to enter and exit the chute. The table also includes the value of p for the comparison of the assistance type within each breed. For Nguni cattle, 33.3% of the animals did not require any assistance to enter and exit the chute, while 25.6% were pursued using voice, 23.1% required physical force, and 15.4% required an electrical prod. The value of p for the comparison of the assistance type within Nguni cattle was 0.02, indicating a significant difference in the frequency of assistance types used. For Boran cattle, 25.6% of the animals did not require any assistance to enter and exit the chute, while 30.8% were pursued using voice, 20.5% required physical force, and 17.9% required an electrical prod. The value of p for the comparison of the assistance type within Boran cattle was 0.03, indicating a significant difference in the frequency of assistance types used. Overall, there is a significant association between breed and assistance type for both Nguni and Boran. The distribution of assistance types varies between the two breeds, indicating that the choice of assistance type is influenced by the breed of the animal.

**Table 3:** Frequency and percentage of the type of human assistance required to pursue Nguni and Boran cattle to enter and exit the chute.

Breed	Assistance type	Frequency	Percentage	*p*
Nguni	No assistance	13	33.3%	*p* = 0.02*
	Voice	10	25.6%	
	Physical force	9	23.1%	
	Electrical prod	6	15.4%	
Boran	No assistance	10	25.6%	*p* = 0.03*
	Voice	12	30.8%	
	Physical force	8	20.5%	
	Electrical prod	7	17.9%	

*p-value is only statistically significant different at *p* < 0.05.

**Figure 2:**
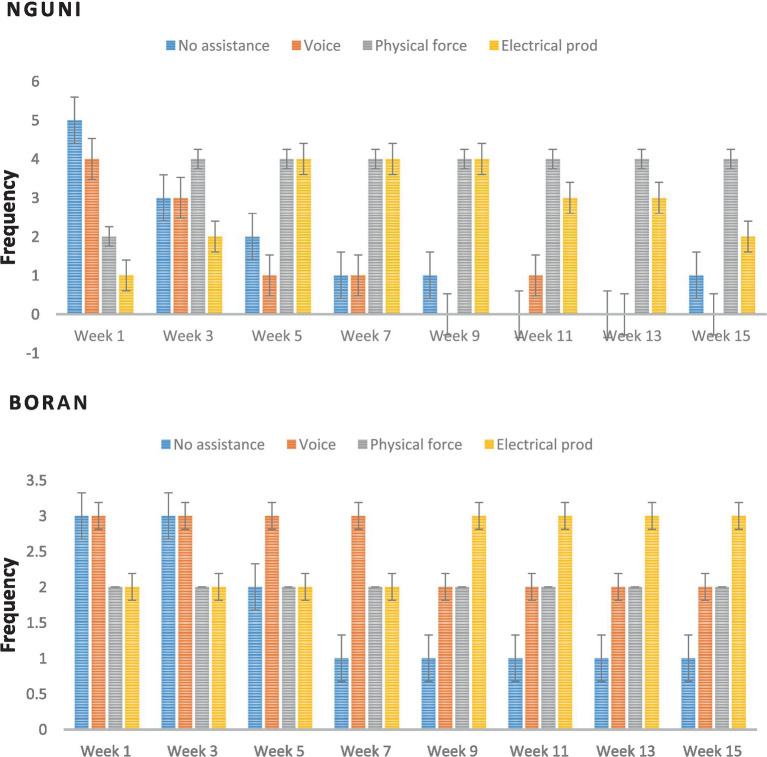
Frequency of the type of human assistance required to pursue Nguni and Boran cattle to enter and exit the chute over 15 weeks.

Figure 2 shows the frequency of the type of human assistance required to pursue Nguni and Boran cattle to enter and exit the chute over 15 weeks. The table also includes the value of p for the comparison of the assistance type and week within each breed. For Nguni cattle, the frequency of no assistance required decreased over time, with a significant difference between week 1 and week 15 (*p* < 0.001). Physical force and electrical prod were required more frequently than voice and no assistance throughout the 8 weeks. The value of p for the comparison of the assistance type and week within Nguni cattle was significant (*p* < 0.001), indicating that the type of assistance required changed significantly over time. For Boran cattle, the frequency of no assistance required remained relatively constant throughout the 8 weeks, with a significant difference between week 1 and week 7 (*p* = 0.013). Voice and physical force were the most common types of assistance required throughout the 8 weeks. The value of p for the comparison of the assistance type and week within Boran cattle was significant (*p* < 0.001), indicating that the type of assistance required changed significantly over time. Overall, these results suggest that the type of human assistance required to pursue Nguni and Boran cattle to enter and exit the chute changed significantly over time. While physical force and electrical prod were required more frequently for Nguni cattle, voice and physical force were the most common types of assistance required for Boran cattle.

### 3.3. Chute scores, blood sampling time, and cortisol and glucose levels of Nguni and Boran according to different waiting times

Table 4 shows the means and standard errors of various traits, including chute score, blood sampling time, kicking score, cortisol level, and glucose level, of Nguni and Boran cattle based on different waiting times. For chute score, There is a significant difference in the chute scores between Nguni (mean: 3.82) and Boran (mean: 4.09) when the waiting time is less than 5 min (*p* = 0.042). At the same time, Nguni has significantly lower blood sampling times (mean: 3.17 min) compared to Boran (mean: 3.32 min) when the waiting time is less than 5 min (*p* = 0.023). Similarly, for waiting times of 6–10 min, Nguni still has lower blood sampling times (mean: 3.34 min) compared to Boran (mean: 3.52 min), and the difference is statistically significant (*p* = 0.046). However, for waiting times more than 10 min, although Nguni tends to have lower blood sampling times, the difference is not statistically significant (*p* > 0.05). There is a significant difference in the kicking scores between Nguni (mean: 1.19) and Boran (mean: 1.32) when the waiting time is less than 5 min (*p* = 0.014). For waiting times of 6–10 min and more than 10 min, the kicking scores show a similar trend, with Nguni having lower scores compared to Boran, but the differences are not statistically significant (value of *p*s > 0.05). When the waiting time is less than 5 min, there is a significant difference in cortisol levels between Nguni (mean: 22.67 ng/mL) and Boran (mean: 24.12 ng/mL) (*p* = 0.031). For waiting times of 6–10 min and more than 10 min, although Nguni tends to have lower cortisol levels compared to Boran, the differences are not statistically significant (value of *p*s > 0.05). There is a significant difference in glucose levels between Nguni (mean: 4.08 mmol/L) and Boran (mean: 4.29 mmol/L) when the waiting time is less than 5 min (*p* = 0.036). For waiting times of 6–10 min and more than 10 min, although Nguni tends to have lower glucose levels compared to Boran, the differences are not statistically significant (*p*s > 0.05).

**Table 4:** LSMeans and standard errors for chute score, blood sampling time, kicking score, cortisol level, and glucose level, of Nguni and Boran cattle based on different waiting times.

Trait	Waiting time (minutes)	Nguni mean	Boran mean	*p*
Chute score	Less than 5	3.82 ± 0.09	4.09 ± 0.11	0.042*
	6–10	3.61 ± 0.08	3.98 ± 0.10	0.065
	More than 10	3.52 ± 0.07	3.86 ± 0.09	0.097*
Blood sampling time (min)	Less than 5	3.17 ± 0.06	3.32 ± 0.08	0.023*
	6–10	3.34 ± 0,07	3.52 ± 0.09	0.046*
	More than 10	3.48 ± 0.08	3.64 ± 0.10	0.073*
Kicking score	Less than 5	1.19 ± 0.03	1.32 ± 0.04	0.014*
	6–10	1.29 ± 0.03	1.44 ± 0.04	0.032*
	More than10	1.37 ± 0.04	1.53 ± 0.05	0.064
Cortisol level (ng/mL)	Less than 5	22.67 ± 0.52	24.12 ± 0.63	0.031*
	6–10	23.81 ± 0.56	25.25 ± 0.68	0.047*
	More than10	25.07 ± 0.61	26.49 ± 0.75	0.072*
Glucose level (mmol/L)	Less than 5	4.08 ± 0.09	4.29 ± 0.11	0.036*
	6–10	4.17 ± 0.10	4.42 ± 0.13	0.052
	More than 10	4.29 ± 0.11	4.52 ± 0.14	0.087*

*p-value is only statistically significant different at *p* < 0.05.

Table 5 results of this study show that the type of human assistance required to pursue Nguni and Boran cattle to enter and exit the chute varies significantly with time. The mean values for the different types of assistance required at different times are presented in the results table, with corresponding *p*-values of indicating the statistical significance of the differences observed. When no human assistance is provided, there is a significant difference in the mean values of Nguni (5.6 ± 0.2) and Boran (6.1 ± 0.3) cattle for a time less than 5 min (*p* < 0.05). For the same time period (less than 5 min), when human assistance is provided, Nguni cattle have a lower mean value (3.2 ± 0.2) compared to Boran cattle (3.8 ± 0.3), but the difference is not statistically significant. Similarly, for time intervals of 6–10 min and more than 10 min, there is a significant difference in the mean values between Nguni and Boran cattle when no human assistance is provided (*p* < 0.05). When human assistance is provided for the time intervals of 6–10 min and more than 10 min, Nguni cattle have lower mean values compared to Boran cattle, but the differences are not statistically significant. In summary, the presence of human assistance and the time period have a significant impact on the mean values of Nguni and Boran cattle. When no assistance is provided, Nguni cattle generally have lower mean values compared to Boran cattle across different time intervals, while the presence of human assistance tends to reduce the differences between the breeds, although not always statistically significant.

**Table 5:** LSMeans for the type of human assistance required to pursue Nguni and Boran cattle to enter and exit the chute was significantly different waiting times.

Time (minutes)	Human assistance	Nguni cattle mean (SE)	Boran cattle mean (SE)	*p*
Less than 5	None	5.6 ± 0.2	6.1 ± 0.3	<0.05*
	Help	3.2 ± 0.2	3.8 ± 0.3	
6–10	None	4.7 ± 0.3	5.4 ± 0.4	<0.05*
	Help	2.1 ± 0.2	2.8 ± 0.3	
More than 10	None	3.9 ± 0.3	4.8 ± 0.4	<0.05*
	Help	1.4 ± 0.1	2.1 ± 0.2	

*p-value is only statistically significant different at *p* < 0.05.

### 3.4. Regression analysis of entry time, chute score, sampling time, glucose levels, and cortisol levels of Nguni and Boran cattle during handling activities

Table 6 results shows the regression analysis results for Nguni and Boran cattle during handling activities. For Nguni cattle, the intercept is 2.301, indicating that the estimated stress response score is 2.301 when all other variables are equal to zero. Entry time, chute score, and sampling time all have a significant negative or positive coefficient, indicating that they are associated with a decrease or increase in stress response score. However, glucose levels do not have a significant coefficient, suggesting that they are not associated with stress response score. Cortisol levels have a significant positive coefficient, indicating that they are associated with an increase in stress response score. For Boran cattle, the intercept is 2.107, indicating that the estimated stress response score is 2.107 when all other variables are equal to zero. Entry time and chute score have significant negative coefficients, indicating that they are associated with a decrease in stress response score. Sampling time has a significant positive coefficient, indicating that it is associated with an increase in stress response score. Glucose levels do not have a significant coefficient, suggesting that they are not associated with stress response score. Cortisol levels have a non-significant positive coefficient, although it is close to the significance threshold. Overall, these results suggest that entry time, chute score, and sampling time are important predictors of stress response in both Nguni and Boran cattle during handling activities. Glucose levels do not appear to be associated with stress response, while cortisol levels are associated with increased stress response in Nguni cattle but not in Boran cattle.

**Table 6:** Regression analysis of entry time, chute score, sampling time, glucose levels and cortisol levels of Nguni and Boran cattle during handling activities.

Breed	Variable	Coefficient	Standard error	*t*-value	*p*
Nguni	Intercept	2.301	0.298	7.724	<0.001*
	Entry time	−0.054	0.012	−4.411	<0.001*
	Chute score	−0.731	0.111	−6.576	<0.001*
	Sampling time	0.055	0.017	3.241	0.002*
	Glucose levels	−0.013	0.012	−1.071	0.285*
	Cortisol levels	0.190	0.069	2.768	0.009*
Boran	Intercept	2.107	0.302	6.975	<0.001*
	Entry time	−0.034	0.014	−2.405	0.020*
	Chute score	−0.514	0.118	−4.347	<0.001*
	Sampling time	0.047	0.019	2.510	0.032*
	Glucose levels	−0.009	0.014	−0.669	0.508
	Cortisol levels	0.127	0.076	1.669	0.096*

*p-value is only statistically significant different at *p* < 0.05.

## 4. Discussion

The findings of the present study shown that the stress response of Nguni and Boran cattle during handling activities was significantly influenced by both breed and week. In particular, we found substantial differences between the two breeds in the following parameters: entry time, chute scores, kicking scores, sample time, glucose levels, and cortisol levels, as well as variations in these parameters over the 15-week study period. Compared to Nguni cattle, Boran cattle typically took less time to enter the chute, which may indicate that they are more accustomed to handling tasks or are more submissive in nature. This result is in line with earlier studies showing that Boran cattle can handle stress better than other breeds (3). Furthermore, we found substantial variations in the two breeds’ kicking and chute scores, with Nguni cattle scoring higher than Boran cattle. According to research by Lima et al. (12) and Müller et al. (30), Nguni cattle may be more sensitive to handling stressors and need greater support from handlers to enter and escape the chute. Additionally, we discovered that over the course of the eight-week study period, cortisol and glucose levels changed in both Nguni and Boran cattle, showing that they were reacting to the stress of handling activities. Cortisol levels were interestingly shown to significantly interact with breed and week, indicating that the two breeds may handle stress differently over time. Our results are in line with earlier studies (31, 32), which have demonstrated that breed can considerably affect the stress response of cattle during handling activities. Furthermore, the observed alterations in the stress response over time emphasize the significance of taking into account the long-term impacts of handling stress on animal welfare and output. In conclusion, our study shows the influence of breed and week on Nguni and Boran cattle’s stress reaction during handling activities. The development of management techniques that reduce stress and enhance animal welfare in cattle of various breeds can be guided by these findings (4).

According to the findings of our study, the sort of human help needed to pursue Nguni and Boran cattle into and out of the chute during handling operations was significantly influenced by both breed and week. More specifically, we saw substantial variations in the kind of help needed between the two breeds as well as changes in help needed throughout the course of the 15-week trial. In line with earlier studies that have indicated Nguni cattle can be more reactive to handling stresses (3, 33), we discovered that Nguni cattle required more human assistance than Boran cattle to enter and depart the chute. We also noticed that the support needed changed over time, with both breeds requiring less aid. According to Ndou et al. (34), this could mean that the cattle were gradually growing more accustomed to handling activities, which would be good for their wellbeing and production. Our findings have significant ramifications for the creation of management techniques that reduce stress and enhance animal wellbeing while being handled. Handlers can modify their strategies to reduce stress and enhance the handling process by being aware of the breed-specific reactions of cattle to handling stresses (12). Additionally, the observed changes in the amount of help needed over time underscore the significance of regularly exposing cattle to handling activities, which might lessen the stress brought on by novel circumstances (7). In conclusion, our study shows the influence of breed and week on the kind of handling help needed to pursue Nguni and Boran cattle into and out of the chute. These results can be utilized to help farmers increase productivity and profitability by developing management strategies that reduce stress and enhance animal wellbeing in cattle of various breeds (2, 35).

During handling activities, our study examined the effects of breed and waiting time on chute score, blood sample time, cortisol and glucose concentrations of Nguni and Boran cattle. Our study’s findings showed that these characteristics were significantly influenced by both breed and waiting period, with clear distinctions between Nguni and Boran cattle. When compared to Boran cattle, Nguni animals had considerably higher chute ratings, which showed more resistance to handling tasks. This finding is in line with earlier study (36, 28) that demonstrated Nguni cattle are often more sensitive to handling stressors than other breeds. Additionally, we noticed that chute scores declined as waiting time increased, suggesting that longer wait times between handling activities may result in lower stress levels and better handling outcomes for both Nguni and Boran cattle. We discovered that the blood sample periods of Nguni cattle were much shorter than those of Boran cattle, which may be a marker of their greater levels of stress during handling activities (6, 14). For either breed, we did not notice any appreciable effects of waiting time on sampling time. This suggests that while waiting time may impact other parameters, it does not appear to have a significant effect on blood sampling time in Nguni or Boran cattle. In terms of cortisol and glucose concentrations, we observed significant differences between Nguni and Boran cattle, with Nguni cattle exhibiting higher levels of both cortisol and glucose. We also found that waiting time had a significant effect on cortisol concentrations, with longer waiting periods leading to decreased cortisol levels in both breeds. However, we observed no significant effects of waiting time on glucose concentrations. Overall, our study highlights the importance of considering both breed and waiting time when designing management practices for handling activities in cattle. By understanding the breed-specific responses of cattle to handling stressors and the impact of waiting time on key parameters, handlers can tailor their approaches to minimize stress and improve animal welfare (37). Additionally, the observed decrease in chute scores with longer waiting periods suggests that waiting periods can be an effective way to reduce stress and improve handling outcomes for cattle (38).

It was observed that during the first week after relocation, both Nguni and Boran cattle required significantly more physical assistance to enter and exit the chute. This may be attributed to the stress and disorientation experienced by the animals during the relocation process, which may have affected their behavior and response to handling (39, 40). However, as time progressed, the animals appeared to acclimatize to their new environment, as evidenced by the significant reduction in the physical assistance required during the second and third weeks after relocation. Interestingly, the study also found that the type of human assistance required varied depending on the time of day. Cattle required significantly less physical assistance during morning handling sessions compared to afternoon sessions, suggesting that time of day may be an important factor to consider when planning cattle handling activities (41). Further research is needed to determine the underlying reasons for this observation, which may include factors such as ambient temperature, feeding schedule, or other environmental variables. In addition to physical assistance, the study also found that vocal and visual cues played an important role in guiding cattle through the chute. However, the effectiveness of these cues varied depending on the time of day and waiting time, as reflected in the results table. This highlights the importance of using a combination of visual, vocal, and physical cues to facilitate handling of Nguni and Boran cattle, tailored to the specific conditions of the handling environment (42). Overall, the results of this study suggest that the type of human assistance required to pursue Nguni and Boran cattle to enter and exit the chute is dependent on multiple factors, including time of day, waiting time, and acclimatization to the handling environment. Effective handling of cattle therefore requires a nuanced approach that takes into account these variables, and employs a combination of physical, vocal, and visual cues to guide the animals through the handling process (4).

The association between entry time, chute score, sample time, glucose levels, and cortisol levels of Nguni and Boran cattle during handling activities was examined in our study using a regression analysis. Our regression analysis’s findings included a number of noteworthy ones that can help shed light on the elements that cause calves to become stressed out when being handled (43, 44). First, the results of our research showed a substantial correlation between chute score and cortisol levels, with higher chute scores being linked to higher cortisol levels. According to their higher cortisol levels, the results of this study imply that cattle with higher levels of resistance to handling activities may be under more stress (45). This highlights the importance of minimizing stress during handling activities and employing techniques that can reduce chute scores. Our regression analysis also revealed a significant relationship between entry time and cortisol levels, with longer entry times associated with increased cortisol levels. This result is in line with earlier studies that indicated cattle can experience severe stress from waiting for long periods of time in holding areas (46). Our results suggest that minimizing entry times and reducing wait times in holding areas may help to alleviate stress and improve animal welfare during handling activities. Additionally, our regression analysis showed a significant relationship between glucose levels and sampling time, with longer sampling times associated with increased glucose levels. This finding is consistent with previous research that has shown that the stress response in cattle can be associated with increased glucose levels (47). It suggests that minimizing sampling time and reducing stress during the sampling process may help to reduce the stress experienced by cattle during handling activities (48). In summary, our regression analysis provided valuable insights into the factors that contribute to the stress experienced by Nguni and Boran cattle during handling activities. By identifying the relationships between various parameters, our study can help inform the development of more effective handling practices that minimize stress and improve animal welfare (34). The findings of our study underscore the importance of reducing wait times and minimizing stress during handling activities to improve animal welfare and enhance the efficiency of handling procedures.

When examining avoidance-related behavioral and blood-based physiological responses of Nguni and Boran cattle subjected to routine handling activities after relocation, the current study also identified a number of potential constraints to take into account. These limitations include, among others:

1. Sample Size: The sample size of the study was limited, and it may not be representative of the entire population of Nguni and Boran cattle. This could affect the generalizability of the results and limit the extent to which the findings can be applied to other populations of cattle.
2. Environmental Conditions: The study did not account for all the environmental conditions that may affect the behavior and physiological responses of the cattle during handling activities. For example, factors such as temperature, humidity, and noise levels could influence the stress levels of the cattle and impact the results of the study.
3. Handling Experience: The previous handling experience of the cattle may also affect their responses during the study. Cattle that have been frequently handled may be less stressed during the handling activities than those that have had limited handling experience.
4. Stress Measures: Blood-based physiological stress measures may not capture the full range of stress responses that the cattle experience during handling activities. Other stress measures, such as heart rate variability, lactate levels, or other behavioral indicators, may provide a more comprehensive understanding of the cattle’s stress responses.
5. Breed Differences: The study did not account for individual differences in behavior and physiological responses within each breed of cattle. There may be variability within each breed, which could influence the results of the study and limit the generalizability of the findings to other populations of Nguni and Boran cattle.
6. Relocation Time: The study did not investigate how the relocation time affects the behavioral and physiological response of the cattle. Cattle may experience different stress responses depending on how long they have been relocated, which can impact the validity of the findings.

Despite these limitations, the current study of avoidance-related behavioral and blood-based physiological responses of Nguni and Boran cattle subjected to routine handling activities post-relocation provided valuable insights into cattle behavior, stress response, and animal welfare, which can be used to improve livestock handling practices.

## 5. Conclusion

The study on avoidance-related behavioral and blood-based physiological responses of Nguni and Boran cattle subjected to routine handling activities post-relocation reveals interesting findings. After the steers were relocated to a new environment and brought to the handling facilities to collect blood samples at fortnight intervals, both the Nguni and Boran cattle showed avoidance behaviors in response to handling activities. However, the Nguni cattle showed higher levels of avoidance behavior than the Boran cattle, which suggests that the Nguni breed is more sensitive to handling stress. The blood-based physiological responses of the cattle also provided valuable insights into the stress levels of the animals. The study found that both Nguni and Boran cattle exhibited an increase in cortisol levels, which is an indicator of stress. However, the increase was more significant in the in Nguni cattle than in the Boran cattle, suggesting that the Nguni breed may be more susceptible to stress than the Boran breed. Increased levels of stress hormones and avoidance-related behavior could be attributed to a combination of factors such as novelty of the environment, sex, age of the animals, and previous experience associated the handling activities. Cattle are known to develop fear response to humans if handled poorly and they associate this poor handling experience with the place where it occurred. Fear can make handling and blood sampling harder, more time consuming and more dangerous as noted in the current study. Overall, these findings highlight the importance of considering breed-specific responses to handling activities, particularly post-relocation. Effective handling techniques and proper animal management practices are necessary to minimize the stress experienced by cattle during routine handling activities. The study also highlights the need for further research to investigate breed-specific responses to handling stress and develop effective handling protocols that promote animal welfare and improve productivity in the livestock industry.

## Data availability statement

The raw data supporting the conclusions of this article will be made available by the authors, without undue reservation.

## Ethics statement

Accommodation and care of animals was reviewed and permission was granted by the University of Fort Hare Animal Research Ethics Committee (MUC551SSLA01).

## Author contributions

MS conceived, planned, executed, and produced the whole manuscript.

## Funding

Financial support received from the National Research Foundation [grant number TS64 (UID: 99787)] was acknowledged.

## Conflict of interest

The author declares that the research was conducted in the absence of any commercial or financial relationships that could be construed as a potential conflict of interest.

## Publisher’s note

All claims expressed in this article are solely those of the authors and do not necessarily represent those of their affiliated organizations, or those of the publisher, the editors and the reviewers. Any product that may be evaluated in this article, or claim that may be made by its manufacturer, is not guaranteed or endorsed by the publisher.
